# Extra-Neural Metastases of Malignant Gliomas: Myth or Reality?

**DOI:** 10.3390/cancers3010461

**Published:** 2011-01-27

**Authors:** Patrick Beauchesne

**Affiliations:** Neuro-Oncology, CHU de NANCY, Hôpital Central, CO n°34, 54035 Nancy Cedex, France; E-Mails: beauchesneP@wanadoo.fr or beaupatt@orange.fr; Tel.: +33-3-8385-1688; Fax: +33-3-8385 2259

**Keywords:** extraneural metastasis, malignant gliomas, radiotherapy, chemotherapy, neurosurgery

## Abstract

Malignant gliomas account for approximately 60% of all primary brain tumors in adults. Prognosis for these patients has not significantly changed in recent years— despite debulking surgery, radiotherapy and cytotoxic chemotherapy—with a median survival of 9–12 months. Virtually no patients are cured of their illness. Malignant gliomas are usually locally invasive tumors, though extra-neural metastases can sometimes occur late in the course of the disease (median of two years). They generally appear after craniotomy although spontaneous metastases have also been reported. The incidence of these metastases from primary intra-cranial malignant gliomas is low; it is estimated at less than 2% of all cases. Extra-neural metastases from gliomas frequently occur late in the course of the disease (median of two years), and generally appear after craniotomy, but spontaneous metastases have also been reported. Malignant glioma metastases usually involve the regional lymph nodes, lungs and pleural cavity, and occasionally the bone and liver. In this review, we present three cases of extra-neural metastasis of malignant gliomas from our department, summarize the main reported cases in literature, and try to understand the mechanisms underlying these systemic metastases.

## 1. Introduction

Malignant gliomas account for approximately 60% of all primary brain tumors in adults, and can be separated into three types—anaplastic astrocytoma (AA), anaplastic oligodendroglioma (AO) and glioblastoma multiforme (GBM) —on the basis of histology [1-4]. GBM is the most common and aggressive primary brain tumor in adults and is characterized by a high rate of local recurrence due to intrinsically radio-resistant tumor cell clones. The standard of care consists of surgical resection of as much of the tumor as is considered to be safe, followed by radio- and chemotherapy, and has been so for many decades [1-4]. However, despite advances in neurosurgery and radiotherapy, prognosis remains dismal. The median survival for patients with newly diagnosed GBM is eight to 15 months while prognosis is slightly better for newly diagnosed AA, with a median survival of 24 to 36 months, and for AO, which gives a median survival of 60 months [1-4]. The recent addition of concomitant and maintenance chemotherapy to radiotherapy in patients with GBM has been shown to prolong median survival by approximately three months and increase the two-year survival rate 2.5 fold [5]. The greatest benefit is achieved in patients having undergone prior tumor resection. Overall survival was 27.2% at two years, 16% at three years, 12.1% at four years, and 9.8% at five years with temozolomide, *versus* 10.9%, 4.4%, 3%, and 1.9% with radiotherapy alone (p < 0.0001). A few patients in favorable prognostic categories survive longer than five years [5].

Malignant gliomas are highly invasive tumors relapsing mainly locally and sometimes spreading along the neuraxis. Extra-neural metastases (ENM) have been reported in medical literature from 1928 but are not frequent [6,7]. Their presence is defined by the criteria of Weiss as follows:

1. The metastatic lesion has to meet the histological characteristics of a central nervous system (CNS) tumor;
2. The clinical course should suggest that the first symptoms were due to the CNS tumor;
3. Complete necropsy should be carried out to exclude any other primary tumor;
4. The morphologic features of the primary tumor and metastatic lesion must be identical [8].

Currently, criteria 1, 3, and 4 are generally accepted for diagnosing ENM [8].

The incidence of ENM from primary intra-cranial malignant gliomas is estimated at less than 2% of cases, but, the leptomeningeal and/or medullary metastasis were not concerned [6,7,9-12]. When they do occur, it is usually late in the disease course (median of two years) [6,7,9-12]. While ENM generally occur after a neurosurgical procedure (craniotomy), spontaneous ENM can develop [6,7,9-12]. Metastases most commonly arise from GBM (41.4%), followed by AA (10.3%) and AO (5.25%) for malignant gliomas [6,7,9-12]. For the other type of tumors, the incidence was of 26.7% for medulloblastomas and of 16.4% for ependymomas [6,7,9-12]. The organs most frequently involved by these ENM are the pleural cavity and/or lung (60%), lymph nodes (51%), bones (31%), and liver (22%) [6,7,9-12]. Involvement of other organs such as skin, heart and bone marrow has also been reported [6,7,9-12]. Ventricular-peritoneal shunt metastases occur generally in the abdomen and the lung [9-12].

To date, the mechanism triggering the spread of ENM from malignant gliomas remains ill-defined. The rationale is the spread of malignant gliomas cells outside the CNS. The fact that the main organs affected by ENM are the lungs and lymph nodes indicate hematological and lymphogeneous routes though pathways along pre-existing cavities such as cerebrospinal routes could be also involved. In this review, we report three cases of ENM of malignant gliomas and attempt to understand the mechanisms underlying these systemic metastases by analyzing reports of ENM from malignant gliomas published in the literature.

## 2. Materials and Methods

### 2.1. Case Report 1

A 54 year-old male complaining of progressive headaches and concentration difficulties for one month, was diagnosed with a glioblastoma (GBM) in the right temporal lobe. An intracranial pressure syndrome and left-sided paresis were reported and a craniotomy with incomplete resection was performed. The patient's clinical status improved and he received a standard cranial irradiation and six courses of etoposide (100 mg/m^2^ - J1J2J3). This resulted in stabilization of the tumor on CT scan and complete clinical response and the patient was able to resume normal activities.

Seven months later, the patient experienced diffuse back pain and an alteration of his clinical status was observed. The back pain, which mainly affected the tenth dorsal vertebra, became increasingly severe and caused insomnia. It was resistant to standard pain-killing drugs and relief was obtained by high doses of morphine. A MIBI scan and a whole-body bone scan revealed pathological lesions involving both the dorsal and dorso-lumbar regions and the pelvis (right iliac bone). Dorso-lumbar spine magnetic resonance imaging (MRI) showed vertebral epidural metastasis of the fifth and tenth dorsal vertebrae. A biopsy sample of the right iliac bone was performed, and an ENM from a GBM was diagnosed (immunoreactive staining with glial fibrillary acidic protein was positive on biopsy). The patient rapidly went on to develop a right-sided paresis and his condition deteriorated dramatically. No aggressive therapy was proposed, and the patient died.

Necropsy revealed a right GBM tumor in the CNS and a metastasis in the left medulla (solid with few microscopic foci of necrosis) but excluded a second primary tumor site. The whole-body autopsy confirmed the vertebral body lesions (fifth and tenth dorsal vertebrae) with epidural mass, the iliac bone metastasis extending to the muscles, and revealed a metastasis in the upper right lobe of the lungs and a lesion in the heart septum.

### 2.2. Case Report 2

**Figure 1.**
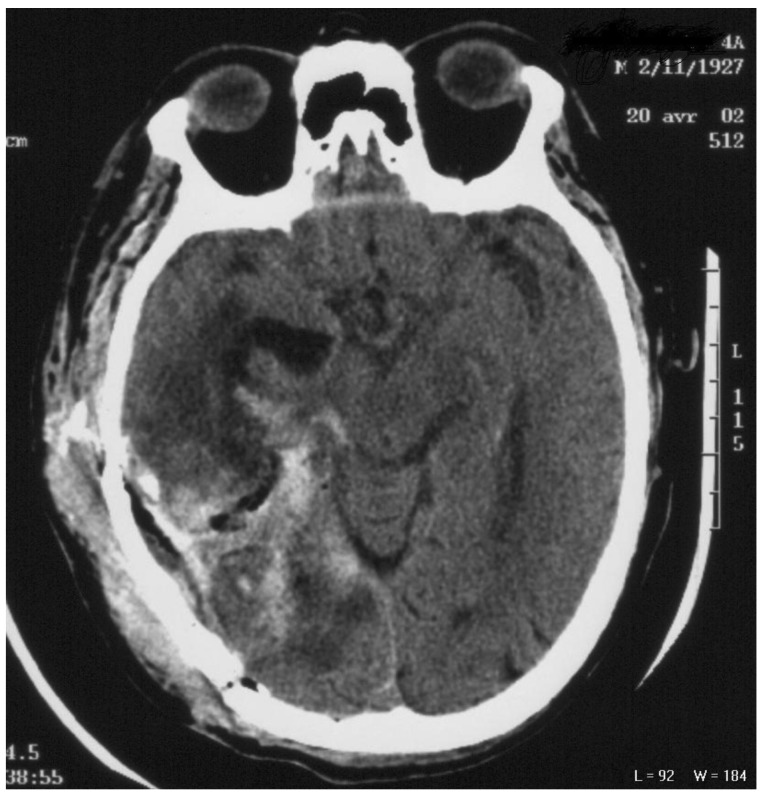
CT-scan axial imaging showing the brain after neurosurgery; a cavity was seen with blood.

A 74 year-old male with a medical history of pulmonary tuberculosis progressively developed agitation and mental confusion over one month. Neurological examination revealed an intracranial pressure syndrome and a left-sided paresis. A cranial MRI displayed a large heterogeneous enhancing mass in the right temporo-occipital lobe. Surprisingly, the biological parameters revealed hyponatremia and a pancytopenia involving the red blood cells and platelets. A craniotomy was performed with an incomplete tumor resection and a GBM was diagnosed (Figure 1). The post-operative course was excellent with improved patient status; the confusion and intracranial pressure syndrome disappeared and the left-sided paresis was mild.

Unfortunately, the pancytopenia dramatically worsened and frequent red cell and platelet transfusions only had a slight effect. A macrophage-activation syndrome and adverse drug reaction were suspected. A lumbar puncture, a myelogram, and a unilateral iliac bone marrow aspirate and biopsy were suspected, in the aim to explicit this unusual pancytopenia and an ENM was diagnosed based on the presence of marked GFAP immunoreactive staining tumor cells. A course of chemotherapy, nitrosourea (fotemustine) was administered, but the patient's status rapidly deteriorated. He experienced back pain involving the cervical and lumbar regions and morphine was required. He continued to receive frequent red cell and platelet transfusions but finally died from a pyrexia pancytopenia syndrome.

**Figure 2.**
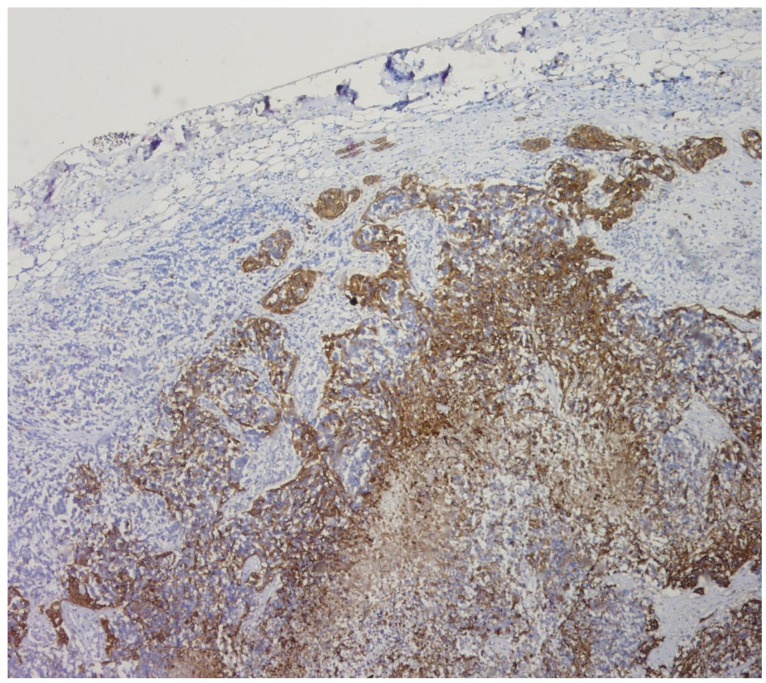
Photomicrograph demonstrating histological findings of the mediastinal lymph node metastasis. Positive immunostaining for glial fibrillary acid protein of lymph node (HES 200).

The autopsy findings ruled out the possibility of a second primary tumor site. Post-mortem examination of the CNS did not reveal any other apart from lesions the initial tumor of the temporo-occipital lobe. The whole-body autopsy also reported diffuse metastasis in the lung, the mediastinal lymph nodes and in the spleen (Figure 2). No other primary tumor was detected.

### 2.3. Case Report 3

A 59-year-old male developed sudden auditory hallucinations which resolved spontaneously and secondary motor aphasia lasting for 10 minutes. This paroxystic aphasia recurred during the following days and the patient also experienced agraphia and alexia. No intracranial pressure syndrome was noted. A cranial MRI was performed and showed a left temporal malignant glioma. A stereotactic biopsy was carried out, and a diagnosis of GBM was retained. The patient was included in a phase II trial testing ultrafractionated cranial radiation therapy: three daily doses of 0.75 Gy delivered at four- hours intervals, five days a week for six consecutive weeks, giving a total dose of 67.5 Gy in 90 fractions. At the end of this therapy, pseudo-progression on cranial MRI was observed, and the patient regained his previous clinical status with moderate alexia only.

**Figure 3.**
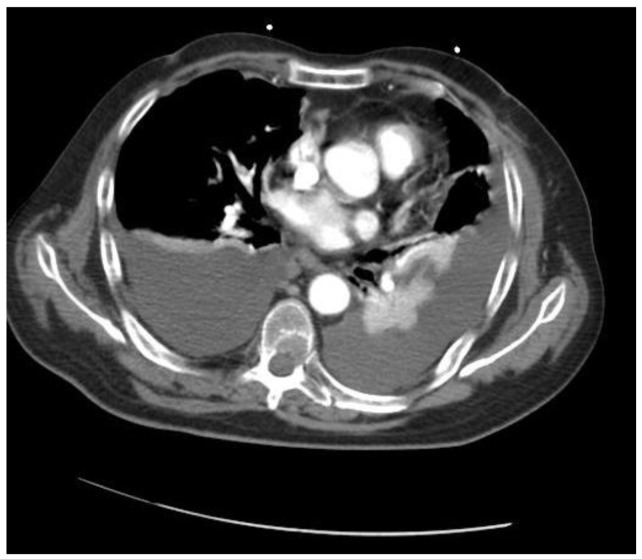
Angio CT-scan showing the metastatic lesions from GBM in the left inferior lobe and in the pleural cavity.

Nine months after the stereotactic biopsy, the patient's clinical status deteriorated; he experienced fatigue, intense back pain involving the dorsolumbar region, worsening alexia. Cranial MRI showed a tumor recurrence and a nitrosourea chemotherapy regimen, fotemustine, was administered (an induction phase of one weekly perfusion of 100 mg/m^2^ for three weeks, and then up to four cycles of adjuvant fotemustine according to the standard regimen of one 100 mg/m^2^ perfusion every 28 days after a four-week break). Despite adjuvant chemotherapy, the patient's status worsened and he was hospitalized in emergency in the neuro-oncology department. Agitation and mental confusion were noted and a secondary cancer of the lung was suspected on thoracic CT scan (bilateral pleural effusion, atelectasis of the left inferior lobe, mediastinal nodes, and diffuse pulmonary lesions) (Figure 3). Symptomatic therapy was administered but no aggressive treatment. The patient finally died.

An autopsy ruled out a secondary primary tumor site. Post-mortem examination of the CNS did not reveal any lesions other than the initial tumor of the left temporal lobe. The whole-body autopsy also reported diffuse metastasis in the lung, the pleural cavity, and the mediastinal lymph nodes and in the liver. No other primary tumor was detected.

### 2.4. Literature Review

Malignant gliomas are unique tumors; many are locally invasive from early on in the disease course and progressively become interspersed with normal brain parenchyma [1-4]. This makes complete resection of malignant gliomas technically impossible [1-4]. They nearly exclusively remain loco-regional tumors, systemic metastases being extremely rare [10-12]. However, malignant glioma cells are able to grow extra-cranially as demonstrated by Zimmermann who transplanted a murine glioma into the pleural and peritoneal cavities [13]. The few cases reports of ENM in literature also clearly demonstrated that malignant gliomas are able to grow outside the CNS [6,7,9-12].

The incidence of ENM is estimated at around 2% [6,7,9-12]. Pasquier *et al.* reported the first large series of ENM including 72 case reports published between 1928 and 1980 [9]. These ENM meet the Weiss criteria 1, 3 and 4, and diagnosis according to histology was: 48 GBM, 10 Gliosarcomas, five Astrocytomas, four mixed gliomas, two spongioblastoma multiforme, two monstrocellular GBM, and one gliomatosis cerebri [9]. The average survival from diagnosis of ENM to death was 18.2 months for the GBM group (2 to > 60 months), which is an unusually high survival for GBM patients [9]. Hoffman and Duffner reported 282 ENM cases of CNS tumors, and focused on the differences between children and adults; ENM most commonly originate from medulloblastoma in children and from gliomas adults [14].

We found a total of 286 cases of ENM from GBM reported in literature; the sex ratio is M/F 2.1; average age at ENM diagnosis 40 years; and the mean survival time 17 months [6,7,9-12, 14-68]. Thirty-five cases of ENM from OA were reported and 20 cases from gliosarcomas [69-88]. The others were came from medulloblastomas, AA and ependymomas [10-12,89,90]. However, some of these cases of ENM did not comply with the Weiss criteria 1, 3 and 4 and necropsy was not routinely performed [8]. Most of the cases (>80%) occurred after a neurosurgical procedure; craniotomy or ventriculo-peritoneal drainage [6,7,9-12,14,15].

Extra-neural metastases usually occur in the lungs and pleural cavity (60%), in the regional lymph nodes (51%), especially in the cervical group, in the skeleton (31%), where vertebral bodies were mainly involved, and in the liver (22%) [6,7,9-12,14-88]. The others sites for metastases are the scalp, the kidney, the orbit, the spleen and the heart [6,7,9-12,14-88]. Bone metastases from malignant gliomas should show lytic or sclerotic features on neuroimaging, and they are usually reported as multiple lesion spread, although one isolated vertebral metastasis has also been reported [11]. Ventriculoperitoneal shunt-associated metastases generally involve the abdomen and lung [6,7,9-12,14,15].

## 3. Mechanisms of Extraneural Metastasis

In 1926, Bailey and Cushing stated that distant metastasis from primitive brain tumors do not exist. In fact, as we have already seen, ENM, though rare, do occur at a frequency of 2% compared with 10% for cerebral metastases from somatic malignant tumors [6,7,9-12,14-88,91]. The pathogenesis of extra-neural dissemination of brain gliomas is not well elucidated though a few hypotheses have been put forward to explain why they are so uncommon: there are no lymphatic vessels in the CNS; the walls of intra-cerebral veins are too thin and collapse before any tumor penetration; the dense nature of the connective tissue encompassing the dural veins; the lack of communicating channels between the intracerebral perivascular space and extracerebral fluid space; and finally, the intermittent nature of communications between subarachnoid space and extracerebral lymphatic vessels [10-12].

Not surprisingly, ENM usually occur after neurosurgery and shunting as previously described by Huang *et al.*; of the 247 cases of ENM they reported, 95.9% occurred after a neurosurgical procedure [15]. Neurosurgery could facilitate the formation of ENM by incomplete closure of the dura or bony defects could create a direct communication between malignant cell tumors and extra-meningeal vessels and the lymphatic channels [92]. Pasquier *et al.* suggested that the negative pressure of intra-operative opened vessels could aspirate tumor cells [9]. Tumor cells could also easily invade fragile vessels during post-operative repair [93].

### 3.1. Local Factors of the Host Organ

Mere access to extra-neural sites is not sufficient to explain the formation of metastasis, which means that local factors present in the host organ could be involved; *i.e.*, growth factors, cytokines, genetic composition of the malignant glioma cell, extra-cellular matrix components, adhesion molecules, enzyme action, cell motility and the cytoskeleton [12]. The extra-cellular matrix proteins (laminin, collagen, and fibronectin) are limited to the basement membrane of the blood vessels. Cerebral tumors produce the following proteins; tenascin, vitronectin, laminin, and hyaluronic acid, but not collagen [12]. The extent to which extra-cellular matrix proteins, cultured with different astrocytoma cell lines, contribute to a permissive environment for tumor invasion has been evaluated in an *in vitro* model [94]. Collagen, fibronectin, and vitronectin were found to be less permissive. Vascular basement membrane collagen should thus be considered as a protective factor against tumor invasion [94]. Due to the absence of connective stroma in the brain parenchyma, brain tumors are not able to select a sub-population of clones which could invade extra-cranial tissues [95].

The inter-cellular interactions determining tumor invasion, are affected by the adhesion molecules. CD44, overexpressed on the surface of gliomas, as well as abnormal integrins expressed by glioma cells seem to facilitate cell invasion [12,96].

Malignant glioma cells overexpress cytokines receptors such as epidermal growth factor (EGF), fibroblast growth factor, platelet-derived growth factor, and interleukin 6 [1-4,97]. The overexpression of these receptors and the response to cytokine stimulation could affect the metastatic potential of malignant glioma cells, in stimulating tumor proliferation and migration.

Tumor cells also secrete enzymes which are involved in tumor invasion by degrading the vascular basement membrane. These enzymes are tissue proteinases such as urokinas-type plasminogen activator, membrane metalloendoprotease and cathepsin B [12]. Malignant glioma cells seem to be unable to secrete this type of enzymes capable of degrading the vascular basement membrane [1,12].

### 3.2. Cerebrospinal Route

Metastases to the cerebrospinal fluid (CSF) are more common with medulloblastomas and oligodendrogliomas. As 500–550 mL of CSF is produced per day; cells entering the CSF could find a rapid route of dissemination. Onda *et al.* have suggested that lesser differentiated tumor cells and a lower rate of glial fibrillary acidic protein (GFAP) expression contribute to a higher tendency to spread into the CSF [98].

### 3.3. Hematogeneous Pathway

Metastasis is a multistep process: detachment of the cell from the primitive tumor; migration in the blood vessels; arrest and adhesion in a target organ site; extravasation into the organ stroma; formation of micro-metastases; and then tumor growth and neoangiogenesis leading to overt tumor formation [10-12]. Craniotomy breaches the CNS innate defense system and can result in tumor cells gaining entry into blood vessels. This hematogeneous route is the main pathway for lung, bone and spleen metastases. Vertebral ENM occur when the metastatic glioma cells enter the Batson plexus (situated in the anterior lumbar cord), and disseminate into the CSF [62]. The Batson plexus could supply blood to the inferior vena cava and to the lumbar and sacreal vertebrae and so facilitate the formation of ENM to lung and liver as well as lumbar and sacral vertebrae [62]. Connections could occur between the meningeal and craniocervical venous system, the latter system being connected to the internal vertebral venous plexus [62]. The internal vertebral venous plexus flows back to anterior and posterior cervical vertebrae; and could be lead to ENM in the body of axis [62].

The blood-brain barrier (BBB) could also play a role in the occurrence of ENM by acting to resist malignant glioma cells migration. It is made of non-fenestrated tightly packed endothelial cells closely connected to astrocyte foot process. Electron studies have revealed that the BBB is disrupted in the tumor area [99]. Malignant glioma cells have been found in the endothelial basement membrane and these cells send out cytoplasmic formations to the vascular lumen [99]. In the vascular lumen, the neoplastic cells are either free or attached to the endothelial lining. Currently, we do not know the exact protein playing the role of a barrier but, laminin, fibronectin, and type IV collagen could be involved. Some unknown substances secreted by the basement membrane could also play a role by down regulating tumor proteases and plasminogen activators [12].

Patients with malignant tumor disease are not usually candidates for organ donation. However, the 2002 annual report of the United Network for Organ Sharing (UNOS) revealed that 0.9% of donors per year die from a CNS tumor in the U.S. [100]. A recent study has evaluated the potential risk of malignancy transmission from CNS tumor organ donors; no cases of malignancy transmission were observed in recipients of 1,220 organs donated by 397 CNS tumor donors [100]. Nevertheless, eight cases of transmission of donor-derived CNS malignancies have been reported since 1987; seven involved a GBM donor and one a medulloblastoma donor [100]. These case reports suggest that malignant glioma cells can be found and transplanted in peripheral organs. Craniotomy preceded organ transplantation in most of the reported cases suggesting that the malignant glioma cells were washed into the blood stream [10].

Ueda *et al.* reported a case of secondary GBM associated with a distant ENM [101]. They carried out a cDNA microarray analysis on both the primary and metastatic tumor (cervical lymph node) and found that IGFBP2 (insulin-like growth factor binding protein-2) was overexpressed in both tumor specimens. DNA-dependent protein kinase (DNA-PK), involved in DNA repair mechanisms, was strongly positive for the first and second operative tumor, and the positive rate decreased in tumor specimens from third and fourth operations [101]. They purport that IGFBP2 probably plays a role in GBM tumor progression, and DNA-PK in the malignant transformation of glioma [101]. A similar pattern of genetic expression was found in the primary and metastatic tumor specimens suggesting that the mechanism of distant ENM was probably due to direct extra-cranial infiltration from the tumor into blood vessels [101].

### 3.4. Lymphogenic Route

It has been postulated that the lack of lymphatic vessels in the CNS constitutes a barrier to tumor spread. However, McComb demonstrated that a significant fraction of CSF drains into CNS lymphatic vessels. Clinical observations, for example nasal congestion or facial swelling following obstruction of CSF shunts, support the concept that CNS lymphatic vessels do actually exist [102]. Therefore, although we have no proof of the existence of these CNS lymphatic vessels, it could explain the high incidence of ENM in cervical or retro-auricular lymph nodes [10-12].

### 3.5. Continuous Spread

Some temporal lobe GBM could spontaneously invade and destroy temporal bone and so lead to the formation of ENM [103]. Intracranial blood vessels and cranial nerves may also facilitate the extension of tumor cells into extra-dural spaces [104].

### 3.6. Genetic Considerations

The specific immunologic behavior of malignant glioma cells could also play a role in EMN spread. In their work, Huang *et al.* successfully transplanted five human GBM xenografts subcutaneously in severe combined immunodeficient (SCID) mice; the take rate was 72 to 100% [15]. Four glioma cell lines developed spontaneous distant metastases; the metastatic activity rate ranged from 0 to 89%. The incidence of this latter property was not different than the nine other human cancer cell lines [15]. Maat *et al.* grafted chemically induced tumor in rats subcutaneously into synergic rats [105]. Fifty-two percent of the transplanted tumors gave rise to distant metastases suggesting that the clinical rarity of ENM is not a fundamental feature of malignant glioma cells [105].

Park *et al.* reported a molecular genetic analysis in six cases of ENM of GBM [106]. DNA from both the primary and metastatic lesions was studied for genetic alterations commonly found in GBM (TP53 mutations, CDKN2A/p16 deletions, EGFR amplification, and allelic loss of 1p, 10q and 19q). Four cases had TP53 mutations and, remarkably, two different TP53 alterations were observed in the primary and metastatic lesions or in the metastatic tumors. This would suggest that metastatic lesions represent the emergence of sub-clones which were not dominant in the brain GBM [106]. The occurrence of ENM from GBM could be influenced by TP53 alterations and differential clonal selection [106].

## 4. Conclusions

Despite their nearly exclusive loco-regional nature and their dismal prognosis, we have seen that malignant gliomas are able to generate ENM. This remains an uncommon event with an incidence estimated at 2% from the literature. For distant ENM to occur malignant glioma tumors interact with their environment; disruption of bonds within the tumor, vascular invasion, arrest in target organ, migration into target and then growth. However, as they are rapidly fatal in most case, the malignant glioma cells rarely have the time to breach the protective barrier of the CNS and thus to develop ENM. Overall, though, the mechanisms underlying the formation of ENM remain unclear and require further and more detailed investigations.

## References

[1] Behin A., Hoang-Xuan K., Carpentier A.F., Delattre J.Y. (2003). Primary brain tumours in adults. Lancet.

[2] Black P.M. (1991). Brain tumors. Part 1. N. Engl. J. Med..

[3] Black PM Brain tumors. Part 2. N. Engl. J. Med..

[4] DeAngelis LM. (2001). Brain tumors. N. Engl. J. Med..

[5] Stupp R., Hegi M., Mason WP., van den Bent MJ., Taphoorn M., Belanger K., Brandes AA., Maroisi C., Bogdahn U., Curschmann J., Janzer RC., Ludwin SL., Gorlia T., Allgeier A., Lacombe D., Cairncross G., Eisenhauer E., Mirimanoff R.O. (2009). Effects of of radiotherapy with concomitant and adjuvant temozolomide versus radiotherapy alone on survival in glioblastoma in a randomized phase III study: 5-year analysis of the EORTC-NCIC trial. Lancet Oncol..

[6] Beauchesne P., Soler C., Mosnier JF. (2000). Diffuse vertebral body metastasis from a glioblastoma multiforme: A technetium-99m sestamibi single-photon emission computerized tomography study. J. Neurosurg..

[7] Didelot A., Taillandier L., Grignon Y., Vespignani H., Beauchesne P. (2006). Concomitant bone marrow metastasis of a glioblastoma multiforme revealed at the diagnosis. Acta Neurochir. (Wien).

[8] Weiss L. (1955). A metastasizing ependymoma of the cauda equine. Cancer.

[9] Pasquier B., Pasquier D., N'Golet A., Meng Hong Pahn., Couderc P. (1980). Extraneural metastases of astrocytomas and glioblastoma. Clinicopathological study of two cases and review of literature. Cancer.

[10] Schweitzer T., Vince GH., Herbold C., Roosen K., Tonn JC. (2001). Extraneural metastasis of primary brain tumors. J. Neurooncol..

[11] Piccirilli M., Brunetto GMF., Rocchi G., Giangaspero F., Salvati M. (2008). Extra-central nervous system metastases from cerebral glioblastoma multiforme in elderly patients. Clinico-pathological remarks on our series of seven cases and critical review of the literature. Tumori.

[12] Subramanian A., Harris A., Piggott K., Schieff C., Bradford R. (2002). Metastasis to and from the central nervous system- the “relatively protected site”. Lancet Oncol..

[13] Zimmermann H.M. (1957). The natural history of intracranial neoplasms with special reference to gliomas. Am. J. Surg..

[14] Hoffman H.J., Duffner PK. (1985). Extraneural metastases of Central Nervous System tumors. Cancer.

[15] Huang P., Allam A., Taghian A., Freeman J., Duffy M., Suit H.D. (1995). Growth and metastatic behavior of five human glioblastomas compared with nine other histological types of human tumor xenografts in SCID mice. J. Neurosurg..

[16] Slowik F., Balogh I. (1980). Extracranial spreading of glioblastoma multiforme. Zentralbl Neurochir..

[17] Mousavi M. (1980). Bone marrow metastasis from glioblastoma multiforme. J. Med. Soc. N. J..

[18] Dietz R., Burger L., Merkel K., Schimrigk K. (1981). Malignant gliomas - glioblastoma multiforme and astrocytomia III-IV with extracranial metastases. Acta Neurochir. (Wien).

[19] Yung W.K., Tepper S.J., Young D.F. (1983). Diffuse bone marrow metastasis by glioblastoma: Premortem diagnosis by peroxidase- antiperoxidase staining for glial fibrillary acidic protein. Ann. Neurol..

[20] Sadik A.R., Port R., Garfinkel B., Bravo J. (1984). Extracranial metastasis of cerebral glioblastoma multiforme. Neurosurgery.

[21] Steinbok P., Dolman C.L., Goldie J.H. (1985). Variation in response to CCNU of glioblastoma multiforme in brain and cervical lymph node. J. Neurosurg..

[21] Wakabayashi T., Yoshida J., Kageyama N., Mutsuga N., Takeuchi Y. (1985). Extraneural metastasis of malignant glioma through a ventriculo-peritoneal shunt: Growth in peritoneal cavity as ascitic form (in Japanese). No Shinkei Geka.

[22] Ogata A., Tashiro K., Abumiya T., Abe H., Matsuno Y., Nakamura K., Kashiwaba T. (1987). Glioblastoma multiforme with extracranial metastases without previous surgery: Demonstration of extracranial metastases by peroxidase antiperoxidase staining and clinicopathological study (in Japanese). No To Shinkei.

[23] Trattnig S., Schindler E., Ungersbock K., Schmidbauer M., Heimberger K., Hubsch P. (1990). Extra-CNS metastases of glioblastoma: CT and MR studies. J. Comput. Assist. Tomogr..

[24] Newton H.B., Rosenblum M.K., Walker R.W. (1992). Extraneural metastases of infratentorial glioblastoma multiforme to the peritoneal cavity. Cancer.

[25] Zappia J.J., Wolf G.T. (1992). Cervical metastatic glioblastoma multiforme. Arch. Otolaryngol. Head Neck Surg..

[26] Malca S.A., Roche P.H., Pellet W. (1993). Secondary localizations of cerebral glioblastoma. Pathogenic and anatomoclinical focus apropos of a case of multiple bone metastases discosed by vertebral involvement. Neurochirurgie.

[27] Chesnut R.M., Abitbol J.J., Chamberlain M., Marshall L.F. (1993). Vertebral collapse with quadraparesis due to metastatic glioblastoma multiforme: Case report and review of the literature. J. Neuro-Oncol..

[28] Gonzales Campora R., Otal Salaverri C., Vazquez Ramirez F., Salguero Villadiego M., Galera Davidson H. (1993). Metastatic glioblastoma multiforme in cervical lymph nodes. Report of a case with diagnosis by fine needle aspiration. Acta Cytol..

[29] Minami T., Kai T., Hirabaru C., Ishii E., Ueda K., Egami H., Takeshita I. (1993). A case of cerebral glioblastoma with extensive cerebrospinal fluid dissemination: Diagnostic value of immunohistochemical examination and MR imaging. Childs Nerv. Syst..

[30] Mihara F., Ikeda M., Rothman M.I., Numaguchi Y., Kristt D. (1994). Vertebral body metastasis of glioblastoma multiforme with epidural mass formation. Contrast-enhanced MRI study. Clin. Imag..

[31] Shuto T., Fujino H., Inodori S., Nakayama S., Satoh H., Ideguchi H., Tashiro Y. (1995). Glioblastoma multiforme with liver metastasis (in Japanese). No To Shinkei.

[32] Granjon O., Lange F., Authier F.J., Lebargy F. (1995). Pulmonary metastases of glioblastoma. Rev. Mal. Respir..

[33] Jonas S., Bechstein W.O., Lemmens H.P., Neuhaus R., Thalmann U., Neuhaus P. (1996). Liver graft- transmitted glioblastoma multiforme. A case report and experience with 13 multiorgan donors suffering from primary cerebral neoplasia. Transpl. Int..

[34] Vural G., Hagmar B., Walaas L. (1996). Extracranial metastasis of glioblastoma multiforme diagnosed by fine-needle aspiration: A report of two cases and a review of the literature. Diagn. Cytopathol..

[35] Wallace C.J., Forsyth P.A., Edwards D.R. (1996). Lymph node metastases from glioblastoma multiforme. AJNR.

[36] Al-Rikabi A.C., Al-Sohaibani M.O., Jamjoom A., Al-Rayess M.M. (1997). Metastatic deposits of a high-grade malignant glioma in cervical lymph nodes diagnosed by fine needle aspiration (FNA) cytology. Case report and literature review. Cytopathology.

[37] Datta C.K., Weinstein J.D., Bland J.E., Brager P.M., Stewart M.A. (1998). A case of cervical lymph node metastasis resulting from glioblastoma multiforme. W. V. Med. J..

[38] Fecteau A.H., Penn I., Hanto D.W. (1998). Peritoneal metastasis of intracranial glioblastoma via a ventriculoperitoneal shunt preventing organ retrieval: Case report and review of the literature. Clin. Transpl..

[39] Greif J., Horovitz M., Marmor S. (1998). Pleuropulmonary metastasis from an intracranial glioblastoma. Lung Cancer.

[40] Hsu E., Keene D., Ventureyra E., Matzinger M.A., Jimenez C., Wang H.S., Grimard L. (1998). Bone marrow metastasis in astrocytic gliomata. J. Neurooncol..

[41] Waite K.J., Wharton S.B., Old S.E., Burnet N.G. (1999). Systemic metastases of glioblastoma multiforme. Clin. Oncol. (R. Coll. Radiol.).

[42] Frappaz D., Mornex F., Saint-Pierre G., Ranchere-Vince D., Jouvet A., Chassagne-Clement C. (1999). Bone metastasis of glioblastoma multiforme confirmed by fine needle biopsy. Acta Neurochir. (Wien).

[43] Houston S.C., Crocker I.R., Brat D.J., Olson J.J. (2000). Extraneural metastatic glioblastoma after interstitial brachytherapy. Int. J. Radiat. Oncol. Biol. Phys..

[44] Widjaja A., Mix H., Golkel C., Flemming P., Egensperger R., Holstein A., Rademaker J., Becker H., Hundt M., Wagner S., Manns M.P. (2000). Uncommon metastasis of a glioblastoma multiforme in liver and spleen. Digestion.

[45] Hübner F., Braun V., Richter H.P. (2001). Case reports of symptomatic metastases in four patients with primary intracranial gliomas. Acta Neurochir..

[46] Laraqui L., Amarti A., Zouaidia F., Maher M., Kettani F., Saidi A. (2001). Pulmonary metastasis from a glioblastoma. A case report. Rev. Pneumol Clin..

[47] Hata N., Katsuta T., Inoue T., Arikawa K., Yano T., Takeshita M., Iwaki T. (2001). Extracranial metastasis of glioblastoma to the lung and heart with a histological resemblance to small cell carcinoma of the lung: An autopsy case (in Japanese). No Shinkei Geka.

[48] Figueroa P., Lupton J.R., Remington T., Olding M., Jones R.V., Sekhar L.N., Sulica V.I. (2002). Cutaneous metastasis from an intracranial glioblastoma multiforme. J. Am. Acad. Dermatol..

[49] Kuhn U., Kohler H.H., Jecker P. (2003). Rare tumors of the parotid gland. Lymphadenoma of a sebaceous gland and extracranial metastasis from glioblastoma. HNO.

[50] Santos A.V., Saraiva P.F., Santiago B. (2003). Extracranial metastasis of glioblastoma multiforme. Acta Med. Port..

[51] Yasuhara T., Tamiya T., Meguro T., Ichikawa T., Sato Y., Date I., Nakashima H., Ohmoto T. (2003). Glioblastoma with metastasis to the spleen. Neurol. Med. Chir. (Tokyo).

[52] Allan R.S. (2004). Scalp metastasis from glioblastoma. J. Neurol. Neurosurg. Psychiatry.

[53] Erdem A., Tun K., Ugur H.C., Erckul S. (2004). Infratemporal and intraorbital metastasis of the glioblastoma multiforme. A case report. Neurochirurgie.

[54] Fabi A., Vidiri A., Carapella C., Pace A., Occhipinti F., Caroli F., Mirri A., Carlini P., Cognetti F. (2004). Bone metastasis from glioblastoma multiforme without central nervous system relapse: A case report. Anticancer Res..

[55] Moon K.S., Jung S., Lee M.C., Kim I.Y., Kim H.W., Lee J.K., Kim T.S. (2004). Metastatic glioblastoma in cervical lymph node after repeated craniotomies: Report of a case with diagnosis by fine needle aspiration. J. Korean Med. Sci..

[56] Bouillot-Eimer S., Loisean H., Vital A. (2005). Subcutaneous tumoral seeding from a glioblastoma following stereotactic biopsy. Clin. Neuropathol..

[57] Chivukula M., Dincer H.E., Biller J.A., Krouwer H.G., Simon G., Shidham V. (2005). FNAB cytology of extra-cranial metastasis of glioblastoma multiforme may resemble a lung primary: A diagnosis pitfall. Cytojournal.

[58] Jain N., Mirakhur M., Flynn P., Choudhari K.A. (2005). Cutaneous metastasis from glioblastoma. Br. J. Neurosurg..

[59] Rajagopalan V., Francois G., El Kamar F.G., Thayaparan R., Grossbard ML. (2005). Bone marrow metastases from glioblasoma multiforme. A case report and review of the literature. J. Neuro-Oncol..

[60] Tuominen H., Lohi J., Maiche A., Tormanen J., Baumann P. (2005). Mediastinal metastasis of glioblastoma multiforme evolving from anaplastic astrocytoma. J. Neuro-Oncol..

[61] Taha M., Almad A., Wharton S., Jellinek D. (2005). Extra-cranial metastasis of glioblastoma multiforme presenting as acute parotitis. Br. J. Neurosurg..

[62] Utsuki S., Tanaka S., Oka H., Iwamoto K., Sagiuchi T., Fuji K. (2005). Glioblastoma multiforme metastasis to the axis. J. Neurosurg..

[63] Chelly I., Mekni A., Ferchichi L., Houissa S., Kchir N., Haouet S., Khaldi M., Zitouna M. (2006). Bone metastasis from glioblastoma: An unsual course. Neurochiurgie.

[64] Yokosuka K., Ishii R., Suzuki Y., Hirano K., Ishii N., Sekihara Y., Hamazaki S., Nishimura H. (2007). Extraneural metastasis of high grade glioma without simultaneous central nervous system recurrence. Neurol. Med. Chir. (Tokyo).

[65] Mentrikoski M., Johnson M.D., Korones D.N., Scott G.A. (2008). Glioblastoma multiforme in skin: A report of 2 cases and review of the literature. Am. J. Dermatopathol..

[66] Templeton A., Hofer S., Töpfer M., Sommacal A., Fretz C., Cerny T., Gillessen S. (2008). Extraneural spread of glioblastoma-report of two cases. Onkologie.

[67] Armstrong T.S., Prabhu S., Aldape K., Hossan B., Kang S., Childress A., Tolentino L., Gilbert M.R. (2010). A case of soft tissue metastasis from glioblastoma and review of the literature. J. Neuro-Oncol..

[68] Zhen L., Yufeng C., Zhenyu S., Lei X. (2010). Multiple extracranial metastases from secondary glioblastoma multiforme: A case report and review of the literature. J. Neurooncol..

[69] Ordonez N.G., Ayala A.G., Leavens M.E. (1981). Extracranial metastases of oligodendroglioma: Report of a case and review of the literature. Neurosurgery.

[70] Dawson T.P. (1997). Pancytopaenia from a disseminated anaplastic oligodendroglioma. Neuropathol. Appl. Neurobiol..

[71] Araki M., Fan J., Haraoka S., Moritake T., Yoshii Y., Watanabe T. (1999). Extraneural metastasis of anaplastic ganglioglioma through a ventriculoperitoneal shunt: A case report. Pathol. Int..

[72] Sharma A., Agarwal A., Sharma M.C., Anand M., Agarwal S., Raina V. (2003). Bone marrow metastasis in anaplastic oligodendroglioma. Int. J. Clin. Pract..

[73] Morrison T., Bibao J.M., Yang G., Perry J.R. (2004). Bony metastases of anaplastic oligodendroglioma respond to temozolomide. Can J. Neurol. Sci..

[74] Al-ali F., Hendon A.J., Liepman M.K., Liepman M.K., Wisniewski J.L., Krinock M.J., Beckman K. (2005). Oligodendroglioma metastatic to bone marrow. AJNR.

[75] Han S.R., Yoon S.W., Yee G.T., Choi C.Y., Lee D.J., Sohn M.J., Chang S.H., Whang C.J. (2008). Extraneural metastases of anaplastic oligodendroglioma. Case Reports/J. Clin. Neurosci.

[76] Zustovich F., Della Puppa A., Scienza R., Anselmi P., Furlan C., Cartei G. (2008). Metastatic oligodendrogliomas: A review of the literature and case report. Acta Neurochir. (Wien).

[77] Volavsek M., Lamovec J., Popovic M. (2009). Extraneural metastases of anaplastic oligodendroglial tumors. Pathol. Res. Pract..

[78] Feigin I.H., Gross S.W. (1955). Sarcoma arising in glioblastoma of the brain. Am. J. Pathol..

[79] Garret R. (1958). Glioblastoma and fibrosarcoma of the brain with extracranial metaastases. Cancer Phil..

[80] Ehrenreich T., Devlin J.F. (1958). A complex of glioblastoma and spindle-cell sarcoma with pulmonary metastases. Arch. Pathol..

[81] Smith D.R., Hardman J.M., Earle K.M. (1969). Contiguous glioblastoma multiforme and fibrosarcoma with extracranial metastasis. Cancer.

[82] Ojeda V.J., Sterrett G.F. (1984). Cerebral gliosarcoma, pulmonary adenoid-cystic carcinoma, and pulmonary metastatic gliosarcoma: Report of an untreated case. Pathology.

[83] Cerame M.A., Guthikonda M., Kohli C.M. (1985). Extraneural metastases in gliosarcoma: A case report and review of the literature. Neurosurgery.

[84] Matsuyama J., Mori T., Hori S., Nakano T., Yamada A. (1989). Gliosarcoma with multiple extracranial metastases. Case report (in Japanese). Neurol. Med. Chir (Tokyo).

[85] Gjerdrum L.M., Bojsen-Moller M. (1999). 61.year-old male with brain tumor and oral, lung, and palpebral masses. Brain Pathol..

[86] Wharton S.B., Whittle I.R., Collie D.A., Bell H.S., Ironside J.W. (2001). Gliosarcoma with areas of primitive neuroepithelial differentiation and extracranial metastasis. Clin. Neuropathol..

[87] Beaumont T.L., Kupsky W.J., Barger G.R., Sloan A.E. (2007). Gliosarcoma with multiple extracranial metastases: Case report and review of the literature. J. Neuro-Oncol..

[88] Maeda D., Miyazama T., Toyooka T., Shima S. (2010). Temporal gliosarcoma with extraneural metastasis. Case report. Neurol. Med. Chir. (Tokyo).

[89] Graf M., Blaeker H., Fotto H. (1999). Extraneural metastasizing ependymoma of the spinal cord. Pathol Oncol. Res..

[90] Kumar P., Rastogi N., Jain M., Chhabra P. (2007). Extraneural metastases in anaplastic ependymoma. J. Cancer Res. Ther.

[91] Bailey P., Cushing H.A. (1926). A Classification of Tumors of the Glioma Group on a Histogenetic Basis With a Correlated Study of Prognosis.

[92] Pang D., Ashmead J.W. (1982). Extraneural metastasis of cerebellar glioblastoma multiforme. Neurosurg..

[93] Gyepes M.T., D'Angio G.J. (1966). Extracranial metastases from central nervous system tumors in children and adolescents. Radiology.

[94] Giese A., Loo M.A., Rief M.D., Tran N., Berens M.E. (1995). Substrates for astrocytoma invasion. Neurosurgery.

[95] Pansera F., Pansera E. (1992). An explanation for the rarity of extra-axial metastases in brain tumors. Med. Hypotheses.

[96] Merzak A., Koocheckpour S., Pilkington G.J. (1994). CD44 mediates human glioma cell adhesion and invasion in vitro. Cancer Res..

[97] Schneider J., Hofman F.M., Apuzzo M.L.J., Hinton D.R. (1992). Cytokines and immunoregulatory molecules in malignant glial neoplasms. J. Neurosurg..

[98] Onda K., Tanaka R., Takahashi H., Takeda N., Ikuta F. (1989). Cerebral glioblastoma with cerebrospinal fluid dissemination: A clinicopathological study of 14 cases examined by complete autopsy. Neurosurgery.

[99] Kung P.C., Bechstein W.O., Bakay L. (1969). Vascular invasion by glioma cells in man: An electron microscopic study. J. Neurosurg..

[100] Armanios M.Y., Grossman S.A., Yang S.C., White B., Perry A., Burger P.C., Orens J.B (2004). Transmission of glioblastoma multiforme following bilateral lung transplantation from an affected donor: Case study and review of the literature. Neuro-Oncol..

[101] Ueda S., Mineta T., Suzuyama K., Furuta M., Shiraishi T., Tabuchi K. (2003). Biologic characterization of a secondary glioblastoma with extracranial progression and systemic metastasis. Neuro-Oncol..

[102] McComb J.G. (1983). Recent researchinto the nature of cerebrospinal fluid formation and absorption. J. Neurosurg..

[103] Nager G.T. (1967). Gliomas involving the temporal bone clinical and pathological aspects. Laryngoscope.

[104] Sanerkin N.G. (1962). Transdural spread of glioblastoma multiforme. J. Pathol. Bacteriol..

[105] Maat B., van Zwieten M.J., van Bekkum D.W., Vecht C. (1979). Transplantability and metastatic potential of chemically induced rat brain tumours. Biomedicine.

[106] Park C.C., Hartmann C., Folkerth R., Loeffler J.S., Wen P.Y., Fine H.A., Black P.M., Shafman T., Louis D.N. (2000). Systemic metastasis in glioblastoma may represent the emergence of neoplastic subclones. J. Neuropathol. Exp. Neurol..

